# Direct medical costs for patients with schizophrenia: a 4-year cohort study from health insurance claims data in Guangzhou city, Southern China

**DOI:** 10.1186/s13033-018-0251-x

**Published:** 2018-11-23

**Authors:** Hui Zhang, Yuming Sun, Donglan Zhang, Chao Zhang, Gang Chen

**Affiliations:** 1 School of Public Health, Sun Yat-sen University, No. 74, Zhongshan 2nd Road, Guangzhou, China; 2 School of Public Health, University of Michigan, 1415 Washington Heights, Ann Arbor, MI 48109 USA; 3 Department of Health Policy and Management, College of Public Health, University of Georgia, 100 Foster Road, Wright Hall 205D, Athens, GA 30602 USA; 4 Business School, Sun Yat-sen University, No. 135, Xingang Xi Road, Guangzhou, China; 5 College of Medicine and Public Health, Flinders University, Adelaide, SA 5041 Australia

**Keywords:** Schizophrenia, Direct medical costs, Cost of illness, Health insurance, China

## Abstract

**Background:**

Schizophrenia is one of the leading public health issues in psychiatry and imposes a heavy financial burden on the healthcare systems. This study aims to report the direct medical costs and the associated factors for patients with schizophrenia in Guangzhou city, Southern China.

**Methods:**

This was a retrospective 4-year cohort study. Data were obtained from urban health insurance claims databases of Guangzhou city, which contains patients’ sociodemographic characteristics, direct medical costs of inpatient and outpatient care. The study cohort (including all the reimbursement claims submitted for schizophrenia inpatient care during November 2010 and October 2014) was identified using the International Classification of Diseases Tenth version (F20). Their outpatient care information was merged from outpatient claims database. Descriptive analysis and the multivariate regression analysis based on Generalized Estimating Equations model were conducted.

**Results:**

A total of 2971 patients were identified in the baseline. The cohort had a mean age of 50.3 years old, 60.6% were male, and 67.0% received medical treatment in the tertiary hospitals. The average annual length of stay was 254.7 days. The average annual total direct medical costs per patient was 41,972.4 Chinese Yuan (CNY) ($6852.5). The inpatient costs remained as the key component of total medical costs. The Urban Employee Basic Medical Insurance enrollees with schizophrenia had higher average costs for hospitalization (CNY42,375.1) than the Urban Resident Basic Medical Insurance enrollees (CNY40,917.3), and had higher reimbursement rate (85.8% and 61.5%). The non-medication treatment costs accounted for the biggest proportion of inpatient costs for both schemes (55.8% and 64.7%). Regression analysis suggested that insurance type, age, hospital levels, and length of stay were significantly associated with inpatient costs of schizophrenia.

**Conclusions:**

The direct annual medical costs of schizophrenia were high and varied by types of insurance in urban China. The findings of this study provide vital information to understand the burden of schizophrenia in China. Results of this study can help decision-makers assess the financial impact of schizophrenia.

## Introduction

Schizophrenia is one of the leading public health issues in psychiatry and is ranked among the top 25 leading causes of disability worldwide [1, 2]. A meta-analysis study estimated the worldwide prevalence of schizophrenia to be 0.54% [3]. In China, a large-scale study estimated a 1-month prevalence rate of 0.78%, with similar rates found in rural (0.80%) and urban (0.72%) areas [4].

Schizophrenia imposed a heavy financial burden on the healthcare systems. A review of cost-of-illness studies for schizophrenia worldwide reported that the direct medical costs of treating schizophrenia accounted for between 1.4 and 3.0% of total national health expenditures [5]. The economic burden of schizophrenia was found to be more than US$60 billion per year in the United States [6]. In China, schizophrenia has been reported to contribute 1.3% of the total burden of disease [7]. The tremendous changes in insurance coverage implemented in the last decade have the potential to improve access to effective health care for patients with schizophrenia and to potentially reduce health-related disability [7].

China expands health insurance coverage to all urban residents with two social health insurance schemes—the Urban Employee Basic Medical Insurance (UEBMI; launched in 1998) and the Urban Resident Basic Medical Insurance (URBMI; launched in 2007) [8]. The UEBMI (which mainly covers the urban employees) and the URBMI (which covers the urban non-employed residents) have different sources of funding, and offered enrollees different benefit packages and levels of financial protection [8]. Except for above two social health insurance schemes, the Chinese government has also implemented a government-funded Medical Care Aid program, which provides additional subsidies for lower income enrollees with specific disease such as schizophrenia [9], so as to reduce the out-of-pocket (OOP) costs for patients from the poorest households. Critical information regarding the per-person annual direct medical costs associated with schizophrenia is needed for health care planning and financing.

Previous studies have evaluated the economic burden and direct medical costs of schizophrenia mostly in developed countries [10–14] and occasionally in developing countries [15, 16], suggesting a large variation in costs across the international cost-of-illness studies for schizophrenia. In the United States, Cloutier et al. [10] used a retrospective cohort and MarketScan Commercial Claims database to assess the direct health care costs of schizophrenia. This study found that the annual direct health care costs per commercially-insured patient with schizophrenia were US$18,090 in 2013 [10]. Another US study using the same claims database found that the mean cost per patient per month for a patient with schizophrenia was US$1806 (US$21,672 yearly) in 2011 [13]. In Germany, Frey [11] used a retrospective cohort and sickness fund claims database to investigate the burden of schizophrenia. Their results showed that the annual cost attributable to schizophrenia was €12,251 per patient from the payers’ perspective in 2008 [11]. Another Germany study used health insurance claims data to examine the cost of schizophrenia and predictors of hospitalization [14]. They reported that the mean costs of stable and unstable patients with schizophrenia were €1605 and €12,864 respectively in 2006 [14]. In Switzerland, Pletscher et al. [12] combined the health insurance claims data with outpatient physician survey to estimate the cost of schizophrenia, and found that the direct medical costs per patient were €9507 in 2012. In addition, there are limited studies that have examined the direct medical costs of schizophrenia in low- and middle-income countries. In Malaysia, Teoh et al. [15] used the data source from medical chart review and suggested that the mean cost per patient was US$6594 in 2015. In Indian, Grover et al. [16] assessed the cost of care for schizophrenia through interview and questionnaires collected from outpatients only, and found that the annual direct medical costs of care was US$274. As found by a systematic review, claims databases were the most commonly used data sources in most direct medical cost estimation for schizophrenia in the high-income countries, while chart review and interview were the main data sources used in low and middle-income countries [17].

Only a few studies examined the direct medical costs of schizophrenia in China [18–20]. Zhai et al. [19] investigated the economic cost of schizophrenia through a cross-sectional survey and found that the per case annual direct costs of schizophrenia was US$862.81 in 2010. Yang et al. [20] analyzed the inpatient costs of psychiatric patients including schizophrenia in Zhejiang Province and discovered that the median cost of hospitalization was US$1539 in 2010. However, these two studies had relatively small sample sizes and were limited to the perspective of one or two hospitals and hospitalized patients only. Wu et al. [18] estimated the direct medical costs for patients with schizophrenia in Tianjin city, Northern China, and reported that the annual mean costs were US$2863 per patient in 2009. But Wu et al.’s study only included patients covered by one insurance scheme (UEBMI), and did not investigate the key drivers of inpatient costs.

Different from previous literature (which either collected data from a selected sample of hospitals or focused on patients with a particular health insurance), this study aims to examine the direct medical costs for patients with schizophrenia and the factors that were associated with the inpatient costs using a 4-year health insurance claims data from the largest city in Southern China. The data in this study covers all patients who were insured with either of the two urban health insurance schemes.

## Materials and methods

### Data source

Data in this study were obtained from the UEBMI and URBMI claims databases of Guangzhou city for the years 2010 through 2014, which contained sociodemographic information, direct medical costs of inpatient and outpatient care based on actual payments to health care providers. Guangzhou city is the capital of Guangdong Province and is one of the most developed cities in Southern China. By 2014, 96.6% of the registered residents were enrolled in the two insurance schemes in Guangzhou city [21]. The detailed reimbursement policies and benefit packages of the UEBMI and URBMI schemes from Guangzhou city in 2014 are summarized in Table 1.

**Table 1:** Comparison of UEBMI and URBMI policies in Guangzhou city in 2014

	UEBMI			URBMI		
Inception year	2002			2008		
Eligible population	Urban employed (employees; retirees)			Urban non-employed (children and full-time students; unemployed adults; elderly residents not covered by the UEBMI scheme)		
Sources of funding	The employers contribute 6% of the employee’s salary whilst the employees contribute 2% Retirees are exempt from premium contribution			Government subsidy (70%) and individual premium (30%) CNY440 to CNY1800 per person per year for residents (including government subsidy)		
Accounts	Medical savings account (including employee contributions and 30% of employer contributions) for outpatient care; social risk-pooling account (70% of employer contributions) for inpatient care and critical (i.e. chronic or fatal diseases including schizophrenia) outpatient care			Social risking-pooling account (all funds) for inpatient care and critical (i.e. chronic or fatal diseases including schizophrenia) outpatient care		
Service package	Comprehensive			Limited		
	*Inpatient*			*Inpatient*		
Benefit packages of social risk-pooling account	Employees	Primary hospitals	90%	Children and students	Primary hospitals	85%
		Secondary hospitals	85%		Secondary hospitals	75%
		Tertiary hospitals	80%		Tertiary hospitals	65%
Reimbursement rate (Inpatient care)	Retirees	Primary hospitals	93%	Unemployed adults and elderly residents	Primary hospitals	85%
		Secondary hospitals	89.5%		Secondary hospitals	70%
		Tertiary hospitals	86%		Tertiary hospitals	55%
Reimbursed ceiling (Inpatient care)	Six times of local employees’ annual average wage per year (CNY445,470)			Six times of local household disposable income per year (CNY228,324)		
	*Critical outpatient*			*Critical outpatient*		
Reimbursement rate (Outpatient care)	Community health centers		85%	Community health centers		70%
	Non-community institutes		65%	Non-community institutes		50%
Reimbursed ceiling (Outpatient care)	CNY150 per person per month			CNY100 per person per month		

Policy information was obtained from Statistical Bulletin of Guangzhou Social Insurance Bureau, and policy documents; Schizophrenia patients are exempt from deductible for hospitalization in Guangzhou

*UEBMI* Urban Employee Basic Medical Insurance scheme, *URBMI* Urban Resident Basic Medical Insurance scheme, *CNY* Chinese Yuan

### Study design

This was a retrospective cohort study designed to estimate the direct medical costs of patients with schizophrenia. We obtained all the reimbursement claims submitted for inpatient care during November 2010 and October 2014 using the International Classification of Diseases Tenth version (ICD-10) (F20). The cohort was identified using the inpatient claims database that included the records of all insured schizophrenia patients who were admitted to hospitals in Guangzhou between November 1, 2010 and October 31, 2011. Their outpatient care information was then merged from the outpatient claims database. Index diagnosis date for each patient was defined as the first observed primary diagnosis of schizophrenia during November 1, 2010 through October 31, 2011, and the baseline period was defined as the first 12 months following index diagnosis date. The first/second/third follow-up year was the second/third/fourth 12 months following index diagnosis date, respectively. Patients who were under 18 years old were excluded. The final sample included 2971 patients, including 1760 and 1211 patients who were insured with the UEBMI and the URBMI, respectively.

This study adopted the Andersen’s behavioral model [22] as the conceptual framework to identify the predictors of total inpatient costs for patients with schizophrenia. Individual characteristics were identified in terms of: (1) predisposing factors—existing conditions with predispose individuals to use or not use services (e.g. age and gender); (2) enabling factors—conditions that facilitate or impede the use of services (e.g. types of health insurance); and (3) need factors—conditions that healthcare providers recognize as requiring long-term medical treatment (e.g. the severity of disease, which was proxied by the length of stay (LOS) and hospital levels) [22].

### Cost estimation

The health insurance claims databases contained information on the actual direct total medical costs of schizophrenia patients, including the reimbursement from the health insurance scheme (either UEBMI or URBMI), the co-payment from enrollees and the aid received through the government-funded Medical Care Aid Program that were only available for patients who met certain criteria. The total annual direct medical cost (consisting of both inpatient and outpatient costs) was calculated for each patient. Costs of schizophrenia were annual medical costs per capita incurred in the inpatient and outpatient sectors. All costs presented in this study were based on a constant 2014 Chinese Yuan (CNY), adjusted using the Consumer Price Index (CPI) of Guangzhou city [21]. According to the Bank of China, the annual exchange rate between US dollar and CNY in 2014 was: US$1.0 = CNY6.1251.

The inpatient medical costs were categorized as laboratory and diagnostic costs, non-medication treatment costs, medication costs, and bed fees in the health insurance claims database. Laboratory and diagnostic costs referred to the costs of physical examinations and biochemical tests. Medication costs were grouped into traditional Chinese medicine and Western medicine costs. Non-medication treatment costs referred to the costs for any other treatments except for medication, which included blood transfusion costs, surgery fees, anesthesia charges, and costs for medical consumables. Bed fees were the accommodation costs during hospitalization.

Information on patient characteristics (age, gender, type of insurance), hospital levels (primary, secondary, tertiary), and length of stay (LOS) was also obtained from the claims database.

### Statistical analysis

Descriptive statistics [frequency, percentage, mean, and standard deviation (SD)] were calculated for demographic information and costs. Since the medical cost data usually has a skewed distribution, the Kruskal–Wallis test was used to investigate whether the differences in patients’ characteristics of two health insurance schemes were statistically significant. To identify the predictors of total inpatient costs, a popular statistical approach to fit a marginal model for longitudinal data, the Generalized Estimating Equations (GEE) model (specified with gamma family, log link function and unstructured correlation structure), was performed in this study [23]. All statistical calculations were performed using Stata version 12.0 (Stata Corporation, College Station, TX, USA).

## Results

### Patient characteristics

A total of 2971 patients were identified in the baseline period (Table 2). More than half of the patients were male (60.6%). The average age was 50.3 years old (SD = 12.7). Patients in the UEBMI subgroup (n = 1760) were on average aged 52.4 years, while patients in the URBMI subgroup (n = 1211) were slightly younger (47.3 years). Most of the patients received medical treatment in tertiary hospitals (67.0%), and the mean annual LOS was 254.7 days. For this study cohort, there were 2021 patients in the first-year follow-up period, 1901 patients in the second and 1754 patients in the third follow-up periods respectively.

**Table 2:** Baseline patients characteristics

Baseline (2010.11.01–2011.10.31)	Overall	UEBMI	URBMI
No. patients	n = 2971	n = 1760	n = 1211
Gender			
Female	1170.0 (39.4)	736.0 (41.8)	434.0 (35.8)
Male	1801.0 (60.6)	1024.0 (58.2)	777.0 (64.2)
Age (years)			
Mean ± SD	50.3 ± 12.7	52.4 ± 11.8	47.3 ± 13.5
Age group			
18 ≤ age < 30	187.0 (6.3)	66.0 (3.8)	121.0 (10.0)
30 ≤ age < 40	379.0 (12.8)	168.0 (9.5)	211.0 (17.4)
40 ≤ age < 50	847.0 (28.5)	445.0 (25.3)	402.0 (33.2)
50 ≤ age < 60	887.0 (29.9)	625.0 (35.5)	262.0 (21.6)
≥ 60	671.0 (22.6)	456.0 (25.9)	215.0 (17.8)
Hospital level			
Primary	76.0 (2.6)	45.0 (2.6)	31.0 (2.6)
Secondary	905.0 (30.5)	517.0 (29.4)	388.0 (32.0)
Tertiary	1990.0 (67.0)	1198.0 (68.1)	792.0 (65.4)
Length of stay (days)			
Mean ± SD	254.7 ± 135.1	244.9 ± 137.9	268.9 ± 129.8
Days ≤ 100	737.0 (24.8)	482.0 (27.4)	255.0 (21.1)
100 < days ≤ 300	501.0 (16.9)	315.0 (17.9)	186.0 (15.4)
> 300 days	1733.0 (58.3)	963.0 (54.7)	770.0 (63.6)
Follow-up no. patients			
First year (2011.11.01–2012.10.31)	2021	1145	876
Second year (2012.11.01–2013.10.31)	1901	1079	822
Third year (2013.11.01–2014.10.31)	1754	988	766

n(%) for categorical variables and mean ± standard deviation for continuous variables

*UEBMI* Urban Employee Basic Medical Insurance scheme, *URBMI* Urban Resident Basic Medical Insurance scheme, *SD* Standard deviation

### Annual total medical costs of schizophrenia

In the baseline period, the mean annual total direct medical costs per patient was CNY41,972.4 (US$6852.5) and the vast majority was inpatient costs: mean inpatient costs (CNY41,780.9, US$6821.3) versus mean outpatient costs (CNY191.5, US$31.3) (see Table 3). On the other hand, the percentage of OOP expenses out of inpatient costs (14.3%) was much less than the OOP percentage out of outpatient costs (63.6%). During the 3-year follow-up periods, the per capita total medical costs (constant 2014 price) increased to CNY55,934.5 (US$9132.0), CNY55,778.4 (US$9106.5) and CNY56,544.1 (US$9231.5), respectively. The inpatient costs remained as the key component of the total medical costs in the follow-up three periods.

**Table 3:** Annual direct medical costs per patient by insurance type (Four Periods)

Baseline	Overall	UEBMI	URBMI	P-value
	n = 2971	n = 1760	n = 1211	
Total costs				
Mean (CNY)	41,972.4	42,543.1	41,143.0	0.021
SD (CNY)	22,741.3	24,250.8	20,330.0	
Out-of-pocket (%)	13.0%	12.7%	13.5%	
Inpatient costs	n = 2971	n = 1760	n = 1211	
Mean (CNY)	41,780.9	42,375.1	40,917.3	0.018
SD (CNY)	22,909.3	24,390.3	20,547.0	
Out-of-pocket (%)	14.3%	14.0%	14.7%	
Reimbursement (%)	76.1%	85.8%	61.5%	
Aid (%)	9.6%	0.2%	23.8%	
Outpatient costs	n = 329	n = 216	n = 113	
Mean (CNY)	191.5	168.0	225.7	0.031
SD (CNY)	747.5	607.2	913.0	
Out-of-pocket (%)	63.6%	53.9%	74.1%	
Reimbursement (%)	36.4%	46.1%	25.9%	

Follow-up: first year	Overall	UEBMI	URBMI	P-value
	n = 2021	n = 1145	n = 876	
Total costs				
Mean (CNY)	55,934.5	57,369.7	54,058.6	< 0.001
SD (CNY)	19,779.9	21,320.6	17,397.7	
Out-of-pocket (%)	12.3%	10.7%	14.5%	
Inpatient costs	n = 2021	n = 1145	n = 876	
Mean (CNY)	55,887.6	57,332.8	53,998.5	< 0.001
SD (CNY)	19,851.0	21,376.4	17,492.4	
Out-of-pocket (%)	12.9%	11.2%	15.2%	
Reimbursement (%)	77.6%	88.8%	62.1%	
Aid (%)	9.5%	0.0%	22.7%	
Outpatient costs	n = 69	n = 41	n = 28	
Mean (CNY)	47.0	36.9	60.1	0.676
SD (CNY)	339.7	273.3	410.5	
Out-of-pocket (%)	67.0%	55.5%	76.4%	
Reimbursement (%)	32.9%	44.6%	23.6%	

Follow-up: second year	Overall	UEBMI	URBMI	P-value
	n = 1901	n = 1079	n = 822	
Total costs				
Mean (CNY)	55,778.4	57,326.6	53,746.0	< 0.001
SD (CNY)	18,646.0	20,107.1	16,323.7	
Out-of-pocket (%)	8.7%	10.7%	5.9%	
Inpatient costs	n = 1901	n = 1079	n = 822	
Mean (CNY)	55,732.3	57,287.6	53,690.7	< 0.001
SD (CNY)	18,725.5	20,178.4	16,416.4	
Out-of-pocket (%)	8.7%	10.7%	5.9%	
Reimbursement (%)	77.7%	88.9%	62.1%	
Aid (%)	13.6%	0.4%	32.0%	
Outpatient costs	n = 44	n = 22	n = 22	
Mean (CNY)	46.1	39.0	55.3	0.356
SD (CNY)	379.8	360.5	403.8	
Out-of-pocket (%)	68.6%	58.1%	78.4%	
Reimbursement (%)	31.3%	41.9%	21.6%	

Follow-up: third year	Overall	UEBMI	URBMI	P-value
	n = 1754	n = 988	n = 766	
Total costs				
Mean (CNY)	56,544.1	60,163.7	51,875.6	< 0.001
SD (CNY)	21,283.6	22,155.4	19,130.6	
Out-of-pocket (%)	8.2%	9.5%	6.1%	
Inpatient costs (n = 1229)	n = 1754	n = 988	n = 766	
Mean (CNY)	56,502.7	60,145.2	51,804.5	< 0.001
SD (CNY)	21,346.2	22,195.0	19,218.0	
Out-of-pocket (%)	8.1%	9.5%	6.0%	
Reimbursement (%)	77.6%	88.1%	61.9%	
Aid (%)	14.3%	2.4%	32.1%	
Outpatient costs	n = 34	n = 14	n = 20	
Mean (CNY)	41.4	18.4	71.1	0.069
SD (CNY)	480.4	273.5	656.4	
Out-of-pocket (%)	76.6%	57.4%	83.0%	
Reimbursement (%)	23.4%	42.8%	17.0%	

P-values are based on the Mann–Whitney test. All costs were based on a constant 2014 Chinese Yuan (CNY)

*UEBMI* Urban Employee Basic Medical Insurance scheme, *URBMI* Urban Resident Basic Medical Insurance scheme, *SD* standard deviation

When analyzing the annual medical costs by patients’ insurance status, the mean total medical costs for the UEBMI group was higher than the URBMI group during the baseline and 3-year follow-up periods (P < 0.05). Furthermore, in the baseline period the per capita inpatient costs of patients with the UEBMI were higher than those of patients with the URBMI scheme, while the per capita outpatient costs of the UEBMI patients were lower than those of the URBMI patients (P < 0.05). The percentage of reimbursement expenses out of inpatient costs for the UEBMI scheme patients (85.8%) was much higher than the patients with the URBMI scheme (61.5%), because these two insurance schemes had different benefit packages as indicated in Table 1. However, the percentage of government medical aid expenses out of the inpatient costs for the UEBMI patients (0.2%) was much lower than the URBMI patients (23.8%), since most of the poor people who were eligible for the Medical Care Aid Program were enrolled under the URBMI scheme. During the 3-year follow-up periods, the UEBMI scheme patients consistently had higher reimbursement rate, while the URBMI patients always had higher government aid rate, and the percentage of government medical care aid expenses increased annually for both schemes.

### Differences in patients who used inpatient care only and patients who used both inpatient and outpatient care

Among 2971 total patients, there were 2642 schizophrenia patients who used inpatient care only and 329 patients who used both inpatient and outpatient care in the baseline period (see Table 4). The annual total direct medical costs between the patients who used inpatient care only and those who used both inpatient and outpatient care significantly differed according to gender, age group, hospital levels, LOS, and composition (all P < 0.001). The mean total costs for schizophrenia patients who had used inpatient care only (CNY44,144.6) were two times higher than those with both inpatient and outpatient services (CNY24,529.1) (P < 0.01).

**Table 4:** Differences in patients who used inpatient care only and patients who used both inpatient and outpatient care (baseline period)

	Patients used inpatient only		Patients used both inpatient and outpatient		P-value
	Mean	SD	Mean	SD	
No. patients	n = 2642		n = 329		
Baseline (2010.11.01–2011.10.31)					
Gender					< 0.001
Female	40,992.4	23,387.4	24,797.1	16,566.3	
Male	46,079.8	21,526.8	24,259.4	18,365.0	
Age groups					< 0.001
18 ≤ age < 30	27,167.2	19,908.5	24,077.3	15,037.8	
30 ≤ age < 40	33,455.6	23,017.7	25,398.3	16,929.5	
40 ≤ age < 50	41,022.2	21,823.5	21,080.3	15,245.2	
50 ≤ age < 60	48,486.4	21,277.6	26,077.1	19,335.1	
≥ 60	51,116.8	20,017.9	33,827.1	22,827.8	
Insurance types					< 0.001
UEBMI	44,956.4	23,994.1	25,292.3	18,410.2	
URBMI	43,002.9	19,858.8	23,070.2	15,460.2	
Hospital levels					< 0.001
Primary	19,764.9	15,675.1	7008.4	4565.4	
Secondary	32,083.2	16,919.9	18,638.6	12,948.4	
Tertiary	51,104.2	21,765.7	26,117.8	17,952.6	
Length of stay (days)					< 0.001
Days ≤ 100	10,540.1	7063.1	13,868.6	7311.2	
100 < days ≤ 300	33,104.6	14,617.7	37,037.8	13,515.9	
> 300 days	57,186.9	12,403.7	65,797.7	15,486.6	
Composition of inpatient costs					< 0.001
Laboratory and diagnostic costs	5537.7	7175.2	6141.0	6088.2	
Non-medication treatment costs	26,556.5	14,234.1	10,762.6	9275.5	
Medication costs	4775.9	3509.1	2756.9	2660.6	
Bed fees	7274.5	3446.4	3138.9	2511.5	
Inpatient costs	44,144.6	22,385.2	22,799.4	17,640.4	< 0.001
Outpatient costs	–	–	1729.7	1546.2	
Total costs (inpatient + outpatient)	44,144.6	22,385.2	24,529.1	17,461.4	< 0.001

P-values are based on the Kruskal–Wallis test. All costs were based on a constant 2014 Chinese Yuan (CNY)

*UEBMI* Urban Employee Basic Medical Insurance scheme, *URBMI* Urban Resident Basic Medical Insurance scheme, *SD* standard deviation

### Inpatient costs of schizophrenia and composition

**Fig. 1:**
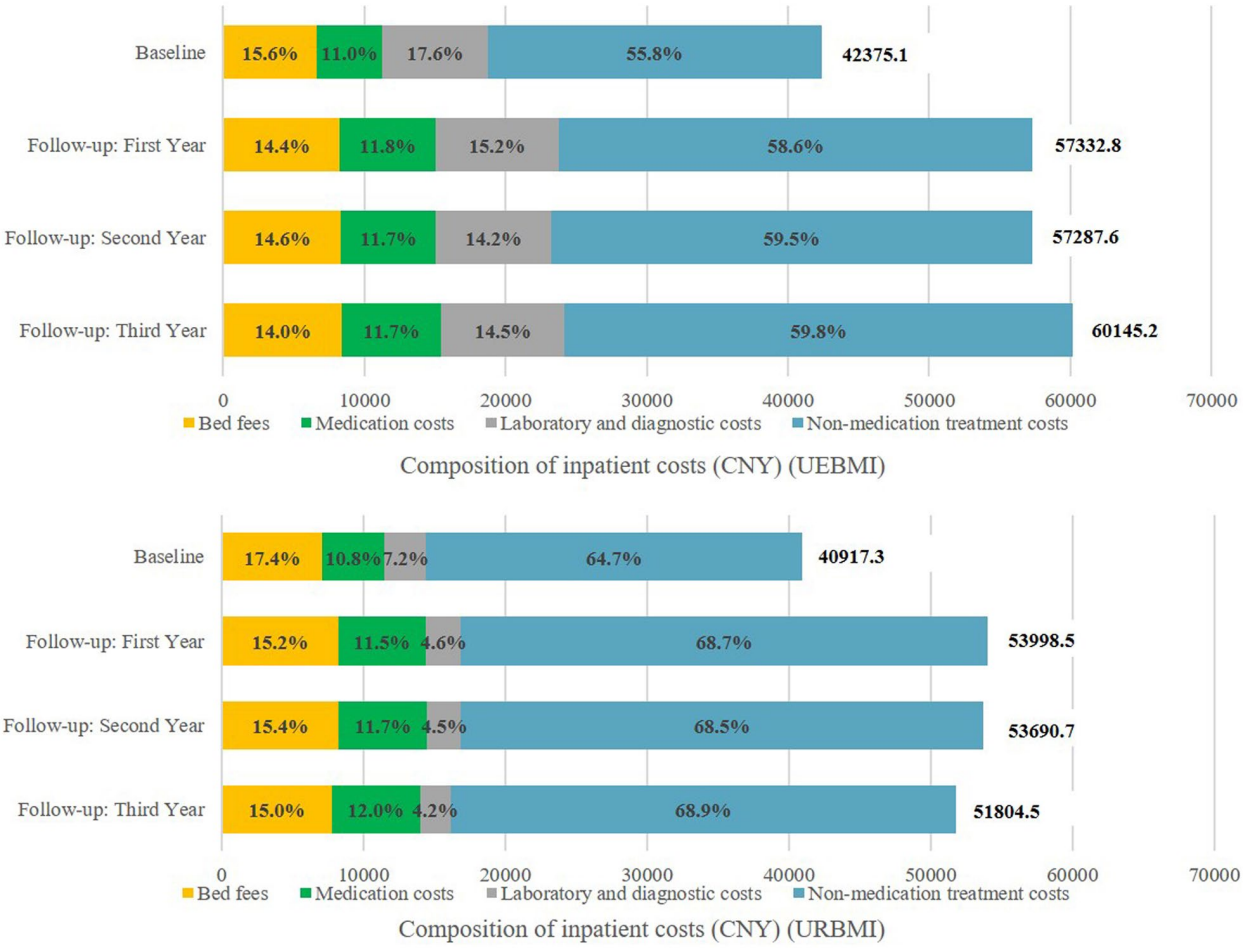
Composition of inpatient costs for 4-year periods: UEBMI and URBMI. All costs were based on a constant 2014 Chinese Yuan (CNY). *UEBMI* Urban Employee-based Basic Medical Insurance scheme, *URBMI* Urban Resident-based Basic Medical Insurance scheme

In the baseline period, inpatient costs between the UEBMI subgroup and URBMI subgroup significantly differed according to gender, age group, hospital levels, LOS, and composition (all P < 0.001) (see Table 5). The mean inpatient costs for schizophrenia patients with the UEBMI (CNY42,375.1) were higher than patients with the URBMI scheme (CNY40,917.3) (Fig. 1). Regarding cost composition, the non-medication treatment costs accounted for the biggest proportion of total inpatient costs for both UEBMI (55.8%) and URBMI (64.7%) schemes. However, the smallest cost component in the UEBMI group was medication costs (11.0%), while the smallest cost component in the URBMI group was laboratory and diagnostic costs (7.2%). During the 3-year follow-up periods, the composition of annual total medical cost remain similar as the baseline period.

**Table 5:** Patients’ characteristics associated with inpatient costs (baseline period)

	Overall		UEBMI		URBMI		P-value
	Mean	SD	Mean	SD	Mean	SD	
No. patients	n = 2971		n = 1760		n = 1211		
Baseline (2010.11.01–2011.10.31)							
Gender							< 0.001
Female	38,472.3	23,410.4	38,225.7	24,643.4	38,890.5	21,176.9	
Male	43,930.2	22,323.1	45,357.4	23,775.3	42,049.3	20,111.6	
Age group							< 0.001
18 ≤ age < 30	25,980.9	19,092.7	23,158.9	20,005.9	27,520.1	18,478.7	
30 ≤ age < 40	31,455.8	22,309.1	28,033.2	24,000.3	34,181.0	20,517.0	
40 ≤ age < 50	38,191.0	22,310.9	36,502.7	24,097.8	40,059.8	20,013.3	
50 ≤ age < 60	46,378.2	22,167.3	46,652.1	23,057.3	45,724.8	19,910.5	
≥ 60	50,470.3	20,403.9	50,308.8	21,999.6	50,812.8	16,559.9	
Hospital levels							< 0.001
Primary	18,457.3	15,537.7	15,274.6	13,706.2	23,077.3	17,052.0	
Secondary	31,244.4	17,065.9	31,131.6	17,767.6	31,394.8	16,104.8	
Tertiary	47,463.3	23,208.1	48,245.2	24,789.2	46,280.6	20,545.9	
Length of stay (days)							< 0.001
Days ≤ 100	10,904.7	7104.7	11,444.1	7441.7	9885.3	6308.5	
100 < days ≤ 300	33,705.9	14,388.3	35,683.3	15,101.7	30,357.1	12,429.8	
> 300 days	57,246.1	12,447.2	60,045.6	13,379.9	53,745.0	10,144.6	
Composition of inpatient costs							< 0.001
Laboratory and diagnostic costs	5604.5	7064.7	7447.3	7778.2	2926.2	4736.8	
Non-medication treatment costs	24,807.5	14,636.9	23,655.7	14,566.6	26,481.5	14,583.1	
Medication costs	4552.4	3483.3	4655.6	3665.6	4402.4	3195.3	
Bed fees	6816.5	3597.7	6616.5	3711.5	7107.2	3406.2	

P-values are based on the Kruskal–Wallis test. All costs were based on a constant 2014 Chinese Yuan (CNY)

*UEBMI* Urban Employee Basic Medical Insurance scheme, *URBMI* Urban Resident Basic Medical Insurance scheme, *SD* standard deviation

### Predictors of inpatient costs

Table 6 shows factors associated with total annual inpatient costs. Regarding the full sample, this study found that insurance type, age, hospital levels, and LOS were significantly associated with inpatient costs of schizophrenia. Compared with patients with the URBMI scheme, the inpatient costs of schizophrenia were CNY5270.0 higher for patients with the UEBMI scheme (P < 0.01). There exists a non-linear relationship between age and total annual inpatient costs, with patients aged 30–40 had the highest inpatient costs among all categories of age. Comparing with the youngest age group (18 ≤ age < 30), inpatient costs for patients aged 30–40 were CNY11,751.0 higher among the UEBMI subgroup and CNY5670.0 higher among the URBMI subgroup (P < 0.01). Gender was a significant factor only among the URBMI group, and male patients incurred CNY1565.8 lower inpatient costs than their female counterparts (P < 0.01). Inpatients staying at tertiary hospitals were found to incur CNY30,330.8 higher inpatient costs among the UEBMI patients and CNY14,701.1 higher among the URBMI patients, compared with patients staying at primary hospitals (P < 0.01). For both subgroups, patients with longer LOS had significantly higher hospitalization costs. Compared with LOS less than 100 days, inpatient costs for the longest LOS group (> 300 days) were CNY89,848.1 higher among the UEBMI patients and CNY84,387.9 higher among the URBMI patients (P < 0.01).

**Table 6:** Factors associated with total inpatient costs **(**GEE Model**)**

	Overall			UEBMI			URBMI		
	n = 2971			n = 1760			n = 1211		
	Coef.	Std. err.	Marginal effect	Coef.	Std. err.	Marginal effect	Coef.	Std. err.	Marginal effect
Follow-up years									
Baseline (reference group)									
First year	0.097***	0.007	4964.3	0.081***	0.010	4277.5	0.120***	0.010	5885.0
Second year	0.087***	0.007	4443.3	0.075***	0.010	3973.3	0.102***	0.010	5027.3
Third year	0.124***	0.008	6356.6	0.126***	0.011	6618.8	0.125***	0.011	6135.1
Age									
18 ≤ age < 30 (reference group)									
30 ≤ age < 40	0.162***	0.030	8280.9	0.223***	0.057	11,751.0	0.115***	0.035	5670.0
40 ≤ age < 50	0.042**	0.018	2148.3	0.074**	0.034	3886.1	0.002	0.018	116.0
50 ≤ age < 60	− 0.001	0.010	− 56.9	− 0.003	0.015	− 172.2	− 0.016	0.012	− 795.9
≥ 60	0.018**	0.008	900.3	0.028***	0.010	1470.9	− 0.011	0.012	− 536.1
Gender									
Female (reference group)									
Male	− 0.016	0.008	− 815.8	− 0.004	0.012	− 203.7	− 0.032***	0.011	− 1565.8
Insurance type									
URBMI (reference group)									
UEBMI	0.103***	0.008	5270.0	–	–	–	–	–	–
Hospital levels									
Primary (reference group)									
Secondary	0.091	0.047	4655.8	0.179**	0.076	9423.8	0.0003	0.047	-14.1
Tertiary	0.447***	0.047	22,894.6	0.576***	0.075	30,330.8	0.299***	0.047	14,701.1
Length of stay (days)									
Days ≤ 100 (reference group)									
100 < days ≤ 300	1.161***	0.019	59,471.0	1.155***	0.025	60,862.5	1.165***	0.029	57,349.4
> 300 days	1.707***	0.017	87,481.9	1.705***	0.022	89,848.1	1.714***	0.025	84,387.9
Wald chi^2^	17,506.97			9853.84			8309.63		
P-value	< 0.0001			< 0.0001			< 0.0001		

The Generalized Estimating Equations (GEE) model estimates are reported in the table

*UEBMI* Urban Employee Basic Medical Insurance scheme, *URBMI* Urban Resident Basic Medical Insurance scheme

*** p < 0.01, ** p < 0.05

## Discussion

This is a retrospective 4-year cohort study conducted with a large schizophrenia sample in Guangzhou city, Southern China. We found that the average annual total direct medical costs for schizophrenia patient was CNY41,972.4 (US$6852.5) per capita in the baseline period, and increased to CNY56,544.1 (US$9231.5) at the end of the 3-year follow-up period. The inpatient costs remained as the key component of the total medical costs in 4 years. The type of insurance, age, higher hospital levels and longer LOS were significantly associated with inpatient costs. This is the first cohort study using sample from the claims database of an entire city to examine the direct medical costs of schizophrenia patients and compare the healthcare costs under two different urban insurance schemes in China.

When comparing our results with findings in other countries, we observed a large variation in costs. Our total direct medical cost (US$6852.5, 2014 price) was much lower than what have been reported in the United States (US$18,090, 2013 price [10]; US$21,672, 2011 price [13]) and European countries, €12,251 (US$17,937, 2008 price) [11] and €12,864 (US$16,141, 2006 price) [14] in Germany, €9507 (US$10,135, 2012 price) [12] in Switzerland, while it was higher than those reported in other Asian countries, such as and US$6594 (2015 price) [15] in Malaysia and US$274 [16] in India (all exchange rates drawn from the OECD Data). Although the international comparison of per person costs for schizophrenia was restricted by different cost components (outpatient or inpatient services), data sources (claims or survey data), and estimation approaches (incidence-based or prevalence-based) included in those studies, the variation lies mostly in the health care systems across different countries.

In this study, the inpatient cost was the largest contributor to the total direct medical costs for patients with schizophrenia, which was consistent with previous studies in other countries [14, 24, 25]. Patients presenting for the first time often showed acute psychotic symptoms that required hospitalization, while treatment for people with repeated relapses was also still predominantly hospital-based across the world [5]. The proportion of costs attributed to inpatient care varied from country to country, depending on the organization of mental health services [5]. Regarding the cost composition, the non-medication treatment costs occupied the biggest proportion of inpatient costs for schizophrenia patients, which included modified electroconvulsive therapy [26], transcranial magnetic stimulation therapy [27], recreational therapy [28], individual psychotherapy [29], and group psychotherapy [30] during hospitalization. The medication costs were found to be the smallest part (10.9%) for inpatients. This percentage of medication costs was found to be lower than 14% in Switzerland [12] and 16% in France [31], but higher than 7% in Netherlands [32]. The medication percentages varied between countries, since differences reflected the structure of services, national pricing policies, the extent and methods of disaggregation of costs, and the market share of the more expensive atypical antipsychotics [5]. When comparing schizophrenia costs to non-schizophrenia costs, a previous study in China indicated that among the schizophrenia patients, schizophrenia-related costs accounted for 62% of all-cause costs per patient [18].

It is difficult to compare the average annual direct medical costs of schizophrenia per patient reported in this study to the other three China-based studies: Wu et al. [18] reported that the mean direct medical costs of patients with schizophrenia were US$2863 (2009 price) in Tianjin city, while US$862.81 (2010 price) was presented by Zhai et al. [19] and US$1539 (2010 price) by Yang et al. [20]. Yang et al’s. [20] study reported only inpatient costs per admission, instead of annual costs per patient. The studies from Wu et al. [18] and Zhai et al. [19] included only 60.8% and 52.2% hospitalized patients, whilst all samples from our study had inpatient care. Thus, our samples were more likely to be relapsed patients with severer conditions, which may incur higher annual expenditures than previous studies.

It is the first time to evaluate the differences in direct medical costs between two urban health insurance schemes. We found that the total direct medical costs and the percentage of reimbursement expenses out of inpatient costs for those covered by the UEBMI scheme were higher than those covered by the URBMI scheme, mostly because the UEBMI had a higher benefit level for its beneficiaries [33]. The panel regression analysis in this study suggested that the inpatient costs of schizophrenia were significantly higher for patients with the UEBMI scheme. There are three possible explanations for this finding. Firstly, those residents who were covered by the UEBMI scheme faced almost equivalent reimbursement rates for services provided by all levels of medical institutions, suggesting a tendency that they would more likely to seek medical treatment at higher level of hospitals and incurred higher expenditures [34]. Secondly, the UEBMI offered a more generous benefits with a higher reimbursement rate for many services, higher annual reimbursement ceiling, and more comprehensive service coverage for its beneficiaries. On the other hand, the URBMI scheme provided neither adequate financial protection nor service coverage for enrolled patients, therefore deter the incentive to use more expensive services among the URBMI beneficiaries [34]. Thirdly, compared with the non-employed patients under the URBMI scheme, the UEBMI patients had higher ability to pay and might be willing to consume additional health services, which were not covered by the health insurance. The current health insurance reform to consolidate the different social health insurance schemes will facilitate the elimination on the disparities in benefit designs across health insurance schemes in financing and reimbursement [8].

Results from the regression analysis found that after controlling for other confounding variables, the association between age and total annual inpatient costs was non-linear, with the 30–40 years old patients had the highest costs. Particularly, the pattern in the differences in medical costs across age categories was quite different between UEBMI patients and URBMI patients. This suggested that the management of costs for schizophrenia patients depending on a category of age might be implemented differently across different insurance schemes. Variations in the costs of schizophrenia tend to be driven by the level of hospital, with patients having medical treatment in tertiary hospitals, being more likely to incur higher inpatient costs [35, 36]. Tertiary hospitals in China are often better equipped. Those advanced diagnostic and medical facilities might cause higher charges in tertiary hospitals than in primary and secondary hospitals in spite of more precise diagnosis and better medical service provided.

The positive association between the LOS and the hospitalization costs of schizophrenia is consistent with previous studies [35, 37]. In the present study, the mean annual LOS per patient was 254.7 days. It was longer than previous studies in United Kingdom (138.9 days) [37], South Korea (128 days) [38], or some other countries (11.1–47.2 days) [39–41]. The LOS of this study was also greater than that (112.1 days) in another China-based study in Tianjin city [18]. It should be noted that it is difficult to directly compare the average annual LOS in this study with above literature since in those studies, the average LOS per admission was reported. Since patients who required a hospital admission associated with a diagnosis of schizophrenia were more likely to have relapsed [14, 42], and the costs of relapse were mainly due to hospital stay [43], our LOS per patient including several admissions would be much longer. To better measure the disease burden of schizophrenia (and taking into account that the schizophrenia patients had multiple admissions within a year), the annual LOS per patient is a better indicator.

In addition, the longer LOS for schizophrenia patients might be due to the reimbursement fee schedule instituted by two urban health insurance schemes in China. In the case of psychiatric hospitalization for the UEBMI and URBMI enrollees, a fixed per diem cost of around US$30 from the insurance fund is paid for the supplies without limitation of LOS [44]. Thus, the hospitals have incentive to extend LOS and increase inpatient admissions [44]. In addition, this study found that most of the schizophrenia patients had used inpatient care exclusively with more acute episodes, could also partly explain the long LOS. The findings of this study suggested that strategies to reduce hospitalization rates and LOS might be an effective method to contain the costs of schizophrenia. Currently, China has limited community psychiatric centers and many hospitals rarely provide services for schizophrenia patients in the outpatient sector [18]. The longer inpatient stays in China may reflect fewer available community-based treatment options [7]. Previous studies in other countries indicated that community-based programs were effective to lower hospitalization rates among psychiatric patients, and they can divert patients from inpatient care to community-based treatment [45, 46]. A survey in 50 low- and middle-income countries suggested that people with schizophrenia in low- and middle-income countries had limited access to specialized mental health services, and inpatient mental health facilities only modestly contributed to overall service accessibility [47]. This study showed that specialized mental health services alone were unable to cope with the burden of schizophrenia in low- and middle-income countries, while primary care services should fill this gap by delivering effective packages of care in collaboration with specialized services [47]. Therefore, efforts to increase usage of community-based treatment programs and psychosocial rehabilitation for schizophrenia patients in China, may reduce the high costs and overuse of medical resources in the hospitals as well as the burden of health insurance funds.

### Limitations

There were some limitations in this study. First, this study only analyzed direct medial costs. The indirect costs due to loss of productivity and family members’ informal care were not examined. Thus, we likely underestimated the total medical expenditures of schizophrenia in China. Second, clinical severity of the disease, disease duration, comorbidities and personal income levels, which were important predictors of costs, were omitted from the analysis because such data were not available in the claims dataset. Third, the study population was limited to urban enrollees under two insurance schemes in one city of China, which cannot generalize to the whole Chinese population. Further studies could consider more associated factors, indirect costs for a more comprehensive evaluation of schizophrenia costs and compare the schizophrenia costs to non-schizophrenia costs to evaluate the impact of health insurance.

## Conclusions

The direct medical costs of schizophrenia were high and varied by types of insurance in China. The findings of this study provide vital information to understand the burden of schizophrenia in China. Such information can also be used by decision makers in program evaluation and health resources allocation.

## Abbreviations

CNY: Chinese Yuan
UEBMI: Urban Employee Basic Medical Insurance
URBMI: Urban Resident Basic Medical Insurance
OOP: out-of-pocket
ICD-10: International Classification of Diseases Tenth version
CPI: Consumer Price Index
LOS: length of stay
SD: standard deviation
GEE: Generalized Estimating Equations
